# Effect of an intensive care unit virtual reality intervention on relatives´ mental health distress: a multicenter, randomized controlled trial

**DOI:** 10.1186/s13054-025-05281-2

**Published:** 2025-02-05

**Authors:** Denzel L. Q. Drop, Johan H. Vlake, Evert-Jan Wils, Jasper Van Bommel, Christian Jung, Denise E. Hilling, O. Joseph Bienvenu, Tim I. M. Korevaar, Anna F. C. Schut, Margo M. C. van Mol, Diederik Gommers, Michel E. van Genderen

**Affiliations:** 1 Department of Adult Intensive Care, Erasmus MC, University Medical Center Rotterdam, Rotterdam, The Netherlands; 2 Department of Intensive Care, Franciscus Gasthuis and Vlietland, Rotterdam, The Netherlands; 3 Medical Faculty and University Hospital of Düsseldorf, Cardiovascular Research Institute Düsseldorf (CARID), Heinrich-Heine University Düsseldorf, 40225 Düsseldorf, Germany; 4 Department of Cardiology, Pulmonology and Vascular Medicine, Medical Faculty, Heinrich-Heine-University Düsseldorf, Düsseldorf, Germany; 5 Department of Surgical Oncology and Gastrointestinal Surgery, Erasmus MC Cancer Institute, University Medical Center Rotterdam, Rotterdam, The Netherlands; 6 Department of Psychiatry and Behavioural Sciences, Johns Hopkins University School of Medicine, Baltimore, MD USA; 7 Department of Internal Medicine, Division of Vascular Medicine and Pharmacology, Erasmus MC, University Medical Center Rotterdam, Rotterdam, The Netherlands; 8 Department of Intensive Care, Ikazia Hospital, Rotterdam, The Netherlands

**Keywords:** Intensive care, Post-intensive Care syndrome, Virtual reality, Quality of life, Mental health

## Abstract

**Background:**

Relatives of intensive care unit (ICU) patients often endure symptoms of post-traumatic stress, anxiety, and depression during and after treatment of a family member’s hospitalization. The aim of this study was to evaluate the effect of ICU-specific virtual reality (ICU-VR) on mental health among relatives, 6 months after patient’s ICU discharge.

**Methods:**

This multicenter, randomized controlled trial included relatives of ICU patients who were assigned to receive either standard care or standard care plus ICU-VR, by randomizing the ICU patients. Relatives were assessed up to 6 months after patient discharge from the ICU for post-traumatic stress, anxiety, depression, quality of life, relatives’ understanding of ICU care, and appreciation of ICU-VR.

**Results:**

One hundred relatives of 81 patients and 89 relatives of 80 patients were randomized to the intervention and control groups, respectively. Relatives’ median age was 48 years and 53% were female. Compared to the control group, relatives who received ICU-VR did not experience a decrease in post-traumatic stress (23% vs. 18%; *p* = 0.99), anxiety (22% vs. 30%; *p* = 0.35), or depression (17% vs. 23%; *p* = 0.44). There was no significant difference between median mental quality of life (50.2 vs. 52.6; *p* = 0.51), physical quality of life (56.1 vs. 54.3; *p* = 0.16), or understanding of ICU care between groups. Patients in the intervention group highly endorsed ICU-VR (90%), favoring it over traditional informational brochures and the majority (82%) stated it improved their understanding of ICU treatment.

**Conclusion:**

ICU-VR did not significantly improve mental health distress symptoms among relatives 6-months after a patient’s discharge. Relatives highly endorsed ICU-VR and self-reported that it improved their understanding of ICU treatment.

**Supplementary Information:**

The online version contains supplementary material available at 10.1186/s13054-025-05281-2.

## Background

Relatives of critically ill patients may experience long-term mental health challenges, including symptoms of post-traumatic stress, anxiety, and depression after their family members’ treatment at the Intensive Care Unit (ICU). These mental health sequalae are collectively referred to as the post-intensive care syndrome-family (PICS-F) and have a negative impact on the health-related quality of life (HRQoL) [1–6].

Previous research focusing on PICS-F interventions did not successfully alleviate symptoms but were of moderate quality [7]. Additionally, society guidelines, such as the Society of Critical Care Medicine, advocate that clear communication and information provision is crucial for improving understanding and reducing stress in relatives, while its lack is perceived as a stressor [8–13]. Recent studies support this hypothesis. Wendlandt et al. reported an association between the lack of information and symptoms of PTSD and De Souza et al. demonstrated that a flexible ICU visitation model in combination with family education was associated with a significant reduction of psychological sequalae [14, 15]. However, because of substantial variability in communication strategies, there is a clear need for a generalizable and adequately tested empirical communication strategy to improve relatives understanding but maybe more importantly also prevent mental health challenges [16, 17].

We previously demonstrated that an ICU-specific informational video, delivered by virtual reality (VR), is feasible and safe and improves information provision and ICU treatment understanding and satisfaction in ICU survivors [18, 19]. Given that families often suffer from a lack of information and communication, ICU-VR can serve as a platform to provide treatment-related and environment-related information in an uniform and structured fashion [9, 20]. This multicenter randomized controlled trial aims to evaluate the effect of ICU-VR on mental health and HRQoL among relatives, 6 months after ICU discharge of their family member. Additionally, we studied the effect of ICU-VR on relatives’ understanding of the ICU environment and procedures and perspectives towards the ICU-VR intervention [21].

## Materials and methods

### Study design and setting

This multicenter, for patient clustered, randomized controlled trial was conducted in three ICU´s in Rotterdam, the Netherlands—including one university hospital and two university-affiliated teaching hospitals—from January 2021 to April 2022. The study protocol was approved by the Medical Ethics Committee of the Erasmus Medical Centre, Rotterdam and of the institutional review boards of the participating centers (protocol number NL73670.078.20, approved on December 14, 2020). The study protocol was previously published [21].

### Participants

Eligible participants were adult (aged ≥ 18 years) first- or second-degree relatives of ICU patients with an expected ICU stay of ≥ 72 h. The primary contact person was approached for participation within 48 h of a patient’s ICU admission by one of the investigators. Multiple relatives per patient could participate per patient and they were permitted to share study-related information with other relatives. Relatives that were unable to understand Dutch, had no smartphone or tablet available, or lacked a formal home address were excluded.

### Stratification and randomization

Participants were cluster-randomized into the intervention group (ICU-VR) or the control group by randomizing the ICU patients. This approach ensured that multiple relatives of the same patient were assigned to the same group, minimizing the risk of cross-contamination between groups. Randomization was stratified according to participating centers and relatives’ ability to visit the hospital as restricted by COVID-19 regulations. The local investigators conducted the randomization using a centralized, internet-based system (Castor Electronic Data Capture (EDC), Amsterdam, the Netherlands). Due to the nature of the intervention, blinding of participants and investigators was not feasible, but the analysis was blinded to the researcher.

### VR intervention

The VR intervention was based on a previous module, developed by an interdisciplinary team including intensivists, ICU nurses, a psychologist, psychiatrist, investigator, and a former ICU patient, with no commercial interests involved for the authors [22]. It comprises several chapters explaining daily aspects of ICU treatment (Fig. S1, Additional File 1) and has been described previously [21]. For the current version, the voice-over was re-recorded to tailor the content specifically for the relatives of ICU patients [21]. The total duration of the VR intervention was 14 min. The point of view for the camera was the field of vision of the mock patient lying in a hospital bed in order to enhance the relative’s empathy with the patient.

### Outcome measures

Our primary outcome is the difference in prevalence and severity of PTSD-, anxiety-, and depression-related symptoms and the difference in HRQoL between the intervention and control group at each follow-up timepoint, with special interest in the difference of PTSD symptoms between the groups at 6 months post-ICU discharge, on which our sample size calculation was based. PTSD symptoms were measured using the Impact of Event Scale-Revised (IES-R), a 22-item questionnaire with a 5-point Likert scale, where scores ≥ 24 indicated mild to severe PTSD [23]. IES-R scores at ICU discharge (T1) are considered as acute stress symptoms, since officially, PTSD needs a duration of at least a month to be diagnosed following the DSM terminology [24]. For symptoms of anxiety and depression, the Hospital Anxiety and Depression Scale (HADS) was used. The HADS consists of 14 items, 7 related to depression and 7 related to anxiety, with a 4-point Likert scale. A sum score ≥ 8 on either the anxiety or depression scale is regarded as present anxiety or depression, respectively [25, 26]. HRQoL was assessed using the Short Form-36 (SF-36) questionnaire, which spans 36 items across eight domains, with each domain score ranging from 0 to 100; higher scores indicate better perceived HRQoL. In addition to these domains, mental (MCS) and physical (PCS) component scores can be calculated, which represent a patient’s mental and physical health state, respectively. These scores are standardized such that the mean is 50 (standard deviation (SD) = 10) for the general population [27–29]. This allows for a consistent comparison of individual scores to the general population's average.

Secondary outcomes were the participants’ perceived quality of care regarding information provision, communication, and their understanding of the ICU environment and procedures. These were assessed using a subset of the Consumer Quality Index-Relatives ICU (CQI-Relatives ICU), tailored to the needs of this study by members of the RATE-XR steering group [21, 30]. The CQI-Relatives ICU was designed according to a robust method developed by the Healthcare Institute of the Netherlands [31, 32]. Perceived stress factors and perspectives on the ICU-VR of participants in the intervention group were assessed using a questionnaire based on the Patient Satisfaction Questionnaire and Family Satisfaction with ICU Care tools, this is described elsewhere [19].

### Study procedures and data collection

As part of routine care, all relatives had access to use an ICU diary and participated in weekly family meetings, or more frequently if clinically necessary, with a dedicated intensivist and ICU nurse [33]. Participants in the intervention group received VR during their first ICU visit via a head-mounted VR display (Oculus Go, Irvine, CA, www.oculus.com/go). This first VR session, which only takes approximately 14 min to limit risk of cybersickness, was carried out by one of the ICU researchers, who was trained to perform the intervention. This researcher guided the participant through the VR process and remained present while the participant watched the VR program. A dedicated ICU nurse or clinician could be contacted in the case participants had questions that were beyond the researcher’s treatment-related knowledge. Afterwards, they could view the VR module as often as desired, from any location, using cardboard VR glasses and a provided access link. Researchers were available for contact in case of technical issues or questions.

Data were collected at five time points: immediately after enrollment at ICU admission (T0), at ICU discharge (T1), and at 1 (T2), 3 (T3), and 6 (T4) months post-ICU discharge of their family member. The HADS and SF-36 questionnaires administered at ICU admission were completed retrospectively, based on the period immediately before ICU admission, while all other outcomes were collected prospectively. An overview of the questionnaires administered at each follow-up time point can be found in the published study protocol [21]. Participants could choose to complete the questionnaires either on paper or online, based on their preference. Non-responders received two reminders for each questionnaire.

### Statistical analysis

Based on previous VR studies, we expected a clinically meaningful Cohen’s *d* effect size of 0.55 in relatives for PTSD at six months post-ICU discharge [18]. Using a two-sided alpha of 0.05, a power of 0.80, and assuming an expected loss-to-follow-up of 20%, we aimed to include relatives of 160 ICU patients.

Continuous variables were presented as median (95% range), categorical variables as frequencies and relative frequencies.

For the primary outcome, differences between groups at each follow-up time point and throughout follow-up in the IES-R sum score, HADS anxiety- and depression score, and the SF-36 MCS and PCS scores were analyzed using mixed effect linear regression models. A random intercept was used for each study site and/or participant based on model comparisons using the Akaiki information criteria. Time and treatment group were added to the model as independent variable and as interaction term. In case of multiple participants for one ICU patient, these participants were considered as clustered, and a random intercept for each cluster was used. Differences in the prevalence of clinically relevant acute stress/PTSD, anxiety, and depression were analyzed using mixed effects logistic regression models. Additionally, a dichotomous composite score (present versus not-present) was calculated to determine the presence of mental health distress (acute stress/PTSD, anxiety, and/or depression, as defined above). If acute stress/PTSD, or anxiety, or depression was present, measured by the IES-R or HADS questionnaires as mentioned before, the composite score for that participant was considered positive. The change in the severity and presence of mental health distress and HRQoL between baseline and follow-up were presented as adjusted mean differences or odds ratios with 95% confidence interval (CI), which were tested for statistical significance using mixed effect linear or logistic regression models.

For the secondary outcome, measured by the CQI-Relatives ICU, differences between groups were analyzed per question using a mixed effect logistic regression model. By combining the numeric values of the answers given, a sum score and subscales for the different sections were calculated for each participant. The association between the intervention and these sum scores were examined using mixed effect linear regression models.

The other outcomes, such as perceived stress factors and perspectives on ICU-VR are described using descriptive statistics and analyzed using mixed-effects linear regression models and mixed-effects logistics regression models, respectively.

Post hoc exploratory analysis were performed to identify potential predictor variables for the development of mental health distress at 1, 3, and 6 months, which was defined as the presence of probable acute stress/PTSD, anxiety, or depression. We first conducted univariate mixed effects logistic regression analyses to identify variables that were associated with the outcomes. Subsequently, all variables with a p-value ≤ 0.20 in the univariate mixed effects regression models were added to the multivariate mixed effects regression models to determine which variables predicted the outcomes, reported as odds ratios (ORs) [95% CI].

All data was gathered using Castor EDC (Castor EDC, Amsterdam, the Netherlands). Analyses were performed using R Statistics (R Foundation for Statistical Computing, Vienna, Austria, 2015). A two-sided *P*-value < 0.05 was considered statistically significant.

## Results

### Baseline characteristics

**Fig. 1:**
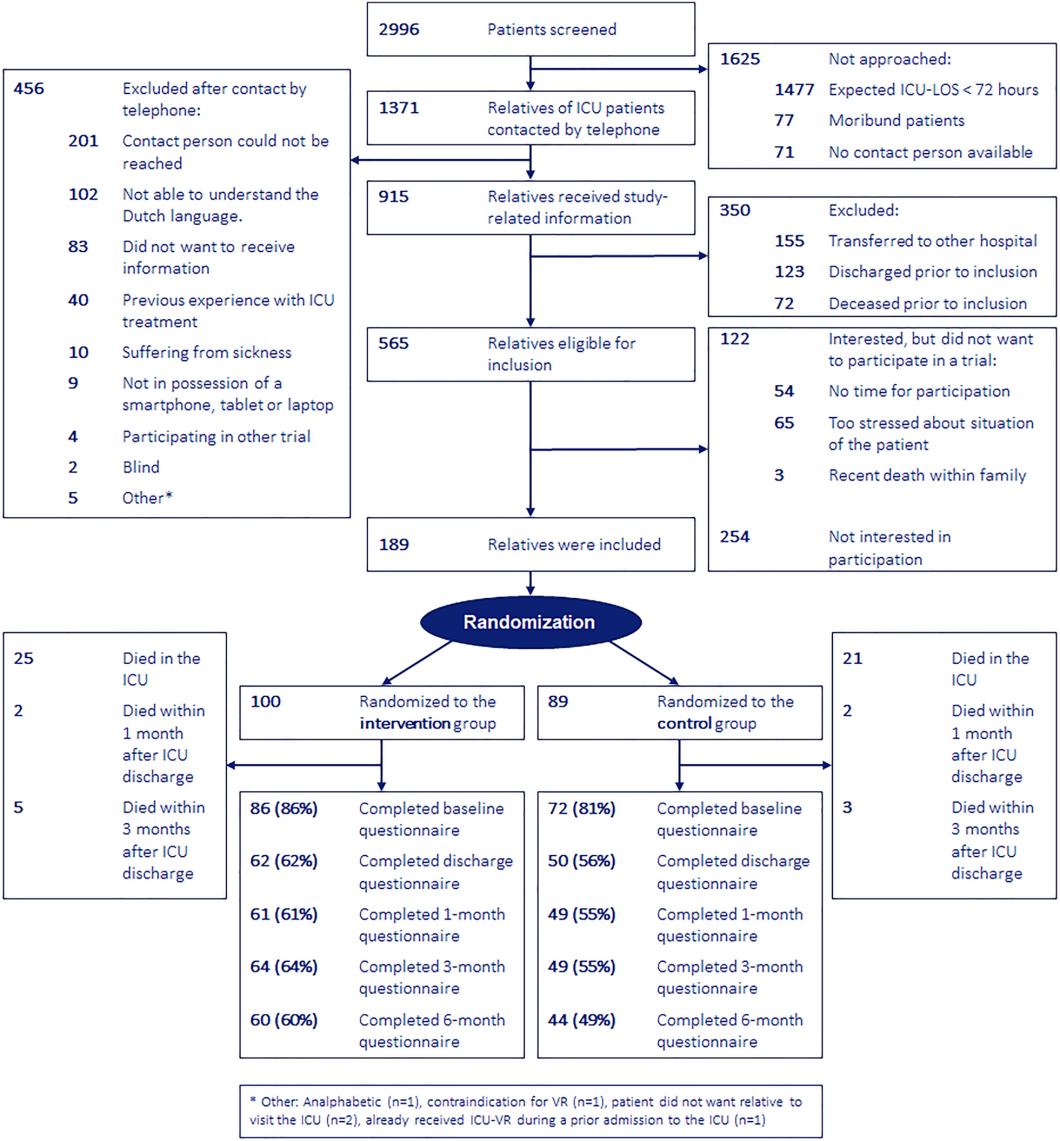
Flowchart of the study. ICU, Intensive care unit; ICU-LOS, Intensive care unit length of stay; VR, virtual reality; ICU-VR, Intensive care unit-specific virtual reality

Between January 2021 and April 2022, a total of 565 relatives of unique ICU patients were eligible for inclusion, of whom 189 relatives of 161 ICU patients consented to participate (inclusion rate 33%). Of these, 100 relatives of 81 patients were randomized into the intervention group, and 89 relatives of 80 patients into the control group. The proportion of completed questionnaires decreased over time, with follow-up rates at six months post-discharge of 60% (60/100) in the intervention group versus 49% (44/89) in the control group (Fig. 1).

The median age of relatives was 48 years (95% range: 23–72), with 53% being female (n = 101). Forty-three percent (n = 69) of relatives were adult children of ICU patients while 39% (n = 62) were spouses or partners. The median age of patients was 62 years (95% range: 25–80) with 35% being female (n = 56). Characteristics of both relatives and patients are presented in Table 1**.**

**Table 1:** Baseline demographics and treatment-related characteristics of participants and patients

Participant characteristics^1^	Intervention group n = 100	Control group n = 89
Age, median (95% range)	45 (20 – 69)	50 (24 – 72)
Gender, female, n (%)	53 (61)	48 (66)
Relation to patient, n (%)		
*Patient is my parent*	40 (46)	29 (40)
*Patient is my spouse*	31 (36)	31 (42)
*Other*^2^	16 (18)	13 (18)
Educational level, n (%)		
*Academic education*	9 (10)	9 (12)
*Higher vocational education*	34 (39)	29 (40)
*Post-secondary vocational education*	32 (37)	23 (32)
*Other*^3^	12 (14)	12 (16)
History of mental illness, yes, n (%)	17 (20)	18 (25)
Allowed to visit hospital, n (%)	87 (87)	80 (90)

Patient characteristics	n = 81	n = 80
Age, median (95% range)	62 (28 – 80)	63 (23 – 78)
Gender, female, n (%)	32 (40)	24 (30)
Reason for admission, n (%)		
*Medical*	51 (63)	47 (59)
*Surgical*	23 (28)	28 (35)
*Trauma*	7 (9)	5 (6)
Confirmed COVID-19, yes, n (%)	30 (37)	22 (28)
Acute Physiology and Chronic Health Evaluation IV score, median (95% range)	59 (5 – 122)	65 (17 – 123)
Received mechanical ventilation, n (%)	72 (89)	75 (94)
*Duration of mechanical ventilation, days, median (95% range)*	9 (2 – 39)	10 (1 – 54)
ICU mortality, n (%)	6 (7)	8 (10)
Length of hospital stay, days, median (95% range)	27 (7 – 104)	31 (8 – 160)
Length of intensive care unit stay, days, median (95% range)	11 (5 – 53)	14 (3 – 100)

^1^Baseline data was retrieved from baseline questionnaire and was not available for 13 [13%] participants in the intervention group and 16 [18%] participants in the control group. Percentages are taken from the available data, except for ‘’Allowed to visit hospital’’, for which the total included sample size is used; ^2^ Patient is my child (intervention: 5 [5%], control: 4 [5%]), patient is my sibling (intervention: 3 [3%], control: 5 [6%]), patient is my cousin (intervention: 1 [1%], control: 2 [2%]), patient is my friend (intervention: 1 [1%], control: 2 [2%], patient is my former partner (intervention: 1 [1%]), patient is my parent in law (intervention: 3 [3%]), patient is my grandparent (intervention: 2 [2%]); ^3^ No education (control: 1 [1%]), secondary vocational education (intervention: 5 [5%], control: 8 [9%]); senior general secondary education (intervention: 7 [7%], control: 3 [3%])

### Mental health distress and quality of life

**Fig. 2:**
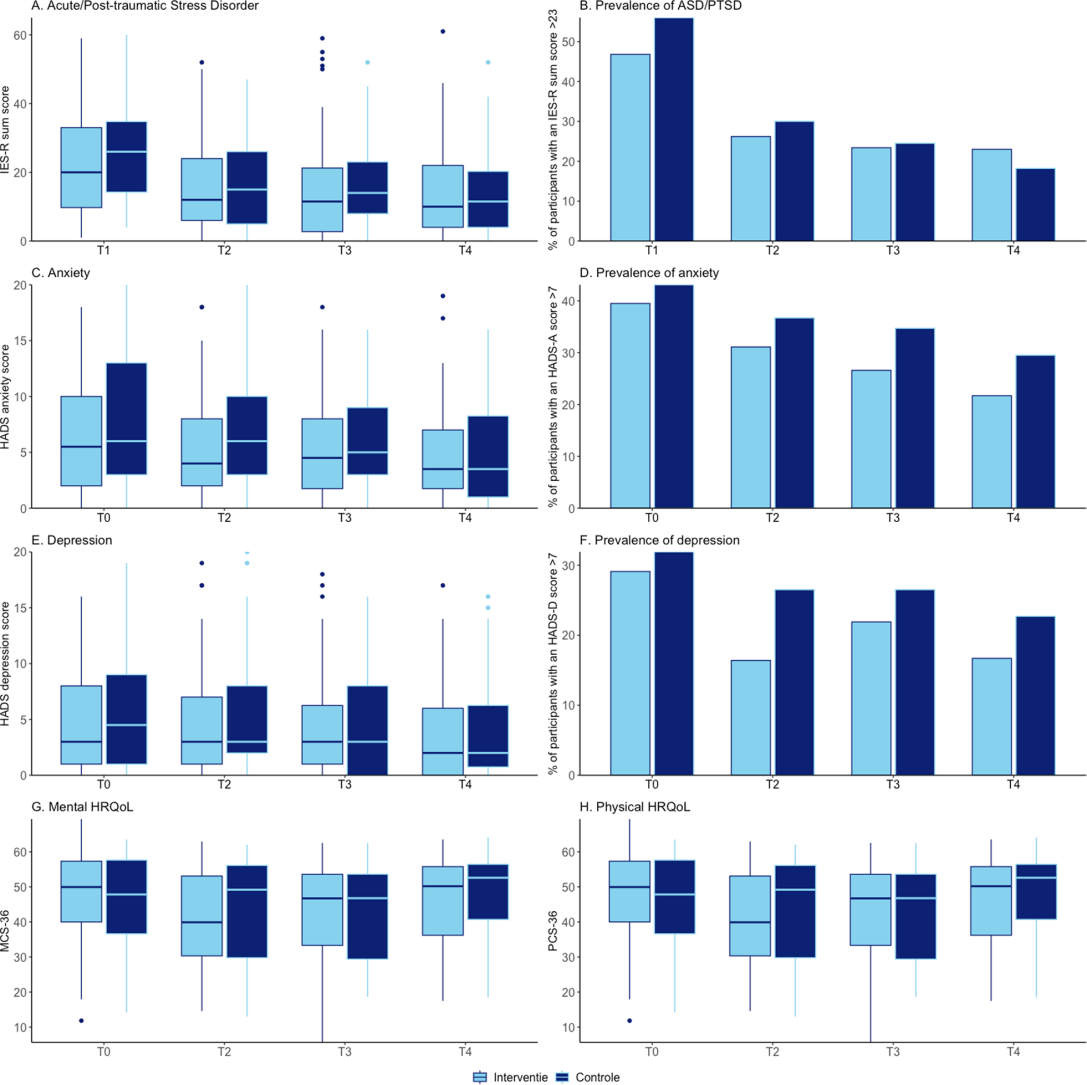
Mental health distress and quality of life. Time points: T0 (retrospective at ICU admission for anxiety, depression, mental HRQoL, and physical HRQoL), T1 (at ICU discharge for ASD/PTSD-related symptoms, T2 (1 month), T3 (3 months), T4 (6 months). IES-R, Impact of Event Scale-Revised; ASD, acute stress disorder; PTSD, Post-traumatic stress disorder; HADS, Hospital Anxiety and Depression Scale; HADS-A, Hospital Anxiety and Depression Scale-Anxiety; HADS-D, Hospital Anxiety and Depression Scale-Depression; HRQoL, health-related quality of life; MCS-36, Mental Component Score -36; PCS-36, Physical Component Score-36

No significant differences were observed between the intervention and control groups at six months (T4) post-ICU discharge for the prevalence of PTSD (23% vs. 18%; p = 0.99), anxiety (22% vs. 30%; p = 0.35), depression (17% vs. 23%; p = 0.44), or the composite score (38% vs. 30%; p = 0.49) (Fig. 2). Similarly, no significant differences were observed between the groups for the severity of PTSD (10 vs. 12; p = 0.90), anxiety (4 vs. 4; p = 0.65), depression (2 vs. 2; p = 0.58), mental HRQoL (50.2 vs. 52.6; p = 0.51), and physical HRQoL (56.1 vs. 54.3; p = 0.16) scores (Fig. 2). Additionally, at 1 month (T2) and 3 months (T3) post ICU discharge, no significant differences were found between groups in terms of the prevalence or severity of PTSD-related symptoms, anxiety, depression, or the mental or physical HRQoL scores.

In the overall cohort, significant reductions in the prevalence of acute stress/PTSD-related symptoms, anxiety, and depression, as well as the severity of acute stress/PTSD-related symptoms and anxiety, were observed between baseline/ICU discharge (T0/T1) and six months post-ICU discharge (T4) (**Tables S1&S2, **Additional File 1). Though, PTSD, anxiety, and depression remained prevalent in 21%, 25%, and 19% of relatives at 6 months (T4) post ICU discharge, respectively. Additionally, the mental composite score shows that 35% (36/104) of relatives suffers from PTSD, anxiety, and/or depression at 6 months (T4) post ICU discharge, compared to 44% (69/158) at ICU admission/discharge (T0/1) (*p* = 0.25) (**Table S1, **Additional File 1**)**. No significant differences in HRQoL outcomes were observed in the overall cohort between baseline (T0) and six months post-ICU discharge (T4) (**Table S3, **Additional File 1).

### Relatives’ understanding and quality of ICU care

**Fig. 3:**
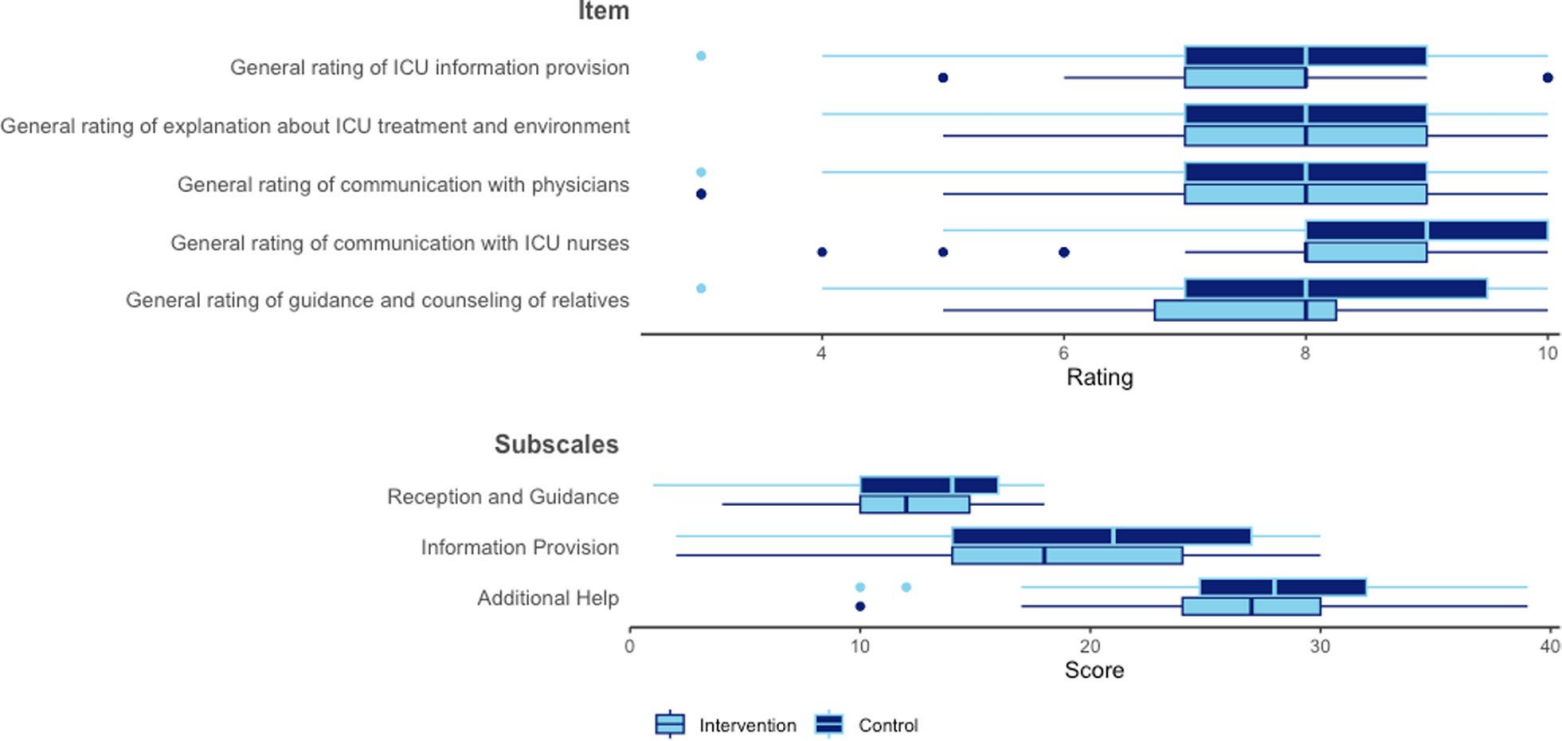
Quality Consumer Index. ICU, Intensive Care Unit; IV, intravenous

Both the intervention and control groups reported similar levels of satisfaction regarding the reception and guidance provided by ICU staff, information provision, additional support, and explanations related to the ICU treatment environment (Fig. 3).

### Experience of ICU-VR

**Fig. 4:**
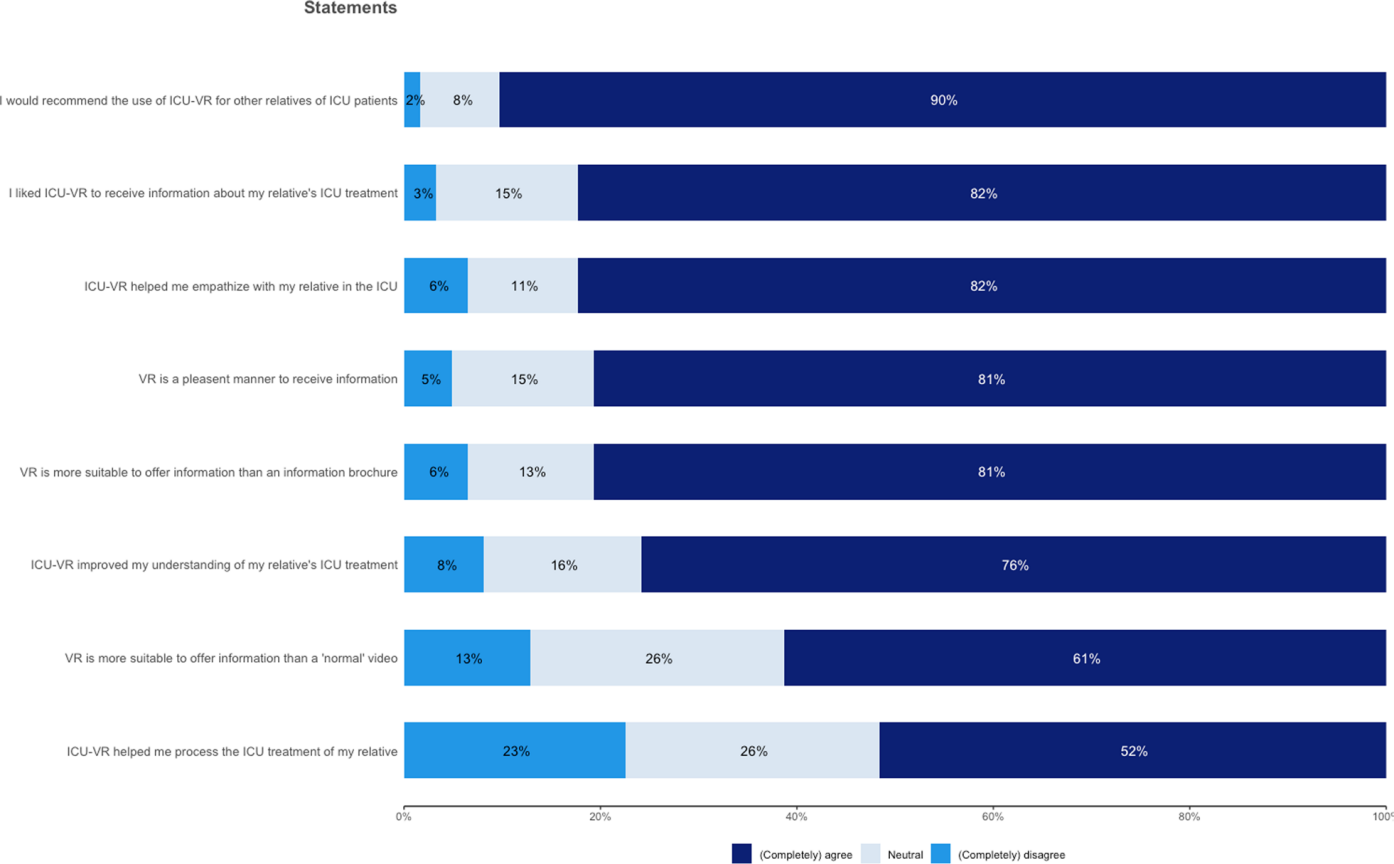
Perspectives on ICU-VR: statements. ICU-VR-Family, Intensive care unit-specific virtual reality-family; ICU, Intensive care unit; VR, virtual reality

ICU-VR was highly endorsed by participants, with 90% recommending the use for other relatives of ICU patients. Additionally, 81% preferred VR over traditional informational brochures, 76% of the participants reported that it improved their understanding of their relative’s ICU treatment, and 52% felt that the VR intervention helped them process their relative´s ICU treatment (Fig. 4). No side effects such as motion sickness were reported.

### Study intervention related outcomes

In the intervention group, 90% (n = 170) of participants received the ICU-VR in the hospital, on average 6 days (95% range: 2–17) after ICU admission. Seven percent (n = 13) received it at home due to visitation restrictions, and 3% (n = 6) did not receive VR because the patient died before the relative could participate. The online VR modules were accessed a total of 203 times, suggesting that, on average, participants watched the intervention an additional two times at home. Over half (58%) showed the intervention to others, primarily family (42%), friends (3%), or both (10%). Furthermore, 61% of participants always used VR goggles to experience the modules at home.

### Risk factors for psychological distress

A post-hoc analysis identified factors that were associated with higher psychological distress. Female relatives were more likely to experience mental health distress at one month (T2) post-ICU discharge (OR: 3.41 [95% CI: 1.22–9.54], *p* = 0.02). Additionally, lower mental HRQoL at ICU admission was associated with increased mental health distress at one month (T2) post-discharge (OR: 4.99 [95% CI: 1.57–15.83], *p* < 0.01) and a history of mental illness was associated with mental health distress at six months (T4) post-discharge (OR: 0.23 [95% CI: 0.07–0.81], *p* = 0.02) (**Table S4A-B, **Additional File 1).

## Discussion

This multicenter patient-clustered randomized trial demonstrated that an ICU-VR intervention for relatives of critically ill patients did not improve mental health distress or HRQoL up to 6 months after ICU discharge. Additionally this trial showed that psychological distress symptoms remained substantially present up to 6 months post-ICU discharge. Lastly, the majority of relatives did report that they felt that ICU-VR improved their understanding of ICU treatment.

Previous research on comparable interventions designed to provide information to relatives of ICU patients showed ambiguous results. Mistraletti et al. observed that informational brochures and websites application only slightly reduced PTSD symptoms, but did not affect anxiety or depression [9]. Similarly, Hoffmann et al. found no effect of an informational website, even though the website was frequently used, reflecting a persistent desire for more information from relatives [34]. While some studies, such as Lautrette et al., found that combining informational brochures with a communication strategy alleviated burden, others, such as Carson et al., observed the opposite effect with similar interventions [8, 35]. Moreover, structured family meetings with an informational brochure have been associated with increased PTSD symptoms [35]. In addition, interventions targeting different mechanisms, such as a standardized family participation program or condolence letters, have also failed to provide substantial improvements in mental health. Condolence letters even exacerbated depression and PTSD-related symptoms [36, 37]. The conclusions of two recent systematic reviews on this subject were at best ambiguous. One concluded that there were no clear benefits from ICU diaries on relatives’ psychological distress, while the other indicated potential reductions in PTSD symptoms [16, 38]. We have not measured or corrected for the use of ICU diaries in our study. However, relatives in both study arms were offered to use digital or hardcopy diaries, according to national guidelines, and therefore we assume a similar effect in both groups.

A common observation among these discussed papers is that RCTs focusing on end-of-life care tend to show more positive results compared to studies conducted in other contexts. This may be a result of the limited control we have over factors post-ICU, which can significantly influence follow-up outcomes. This is a unique challenge for designing and interpreting RCTs in non–end-of-life care settings. Additionally, the broader context outlined by these studies suggests that no single intervention can universally address the psychological impact experienced by ICU relatives and may explain why there is a heterogeneity in the guidance and treatment of post-ICU patients, for example regarding the implementation of aftercare clinics, use of ICU diaries, and application of family participation programs. A more tailored, multifaceted approach, incorporating a combination of interventions like ICU-VR at different stages of the ICU experience may prove more effective. Such an approach is also suggested by others [36, 39, 40]. Moreover, it raises the question of whether the outcome measures used in this and similar studies sufficiently capture the full impact of these interventions. Although many relatives reported positive experiences with the VR intervention and perceived enhanced understanding of the patient’s situation, these subjective benefits were not reflected in the primary outcomes. This discrepancy underscores the limitations of current evaluation metrics in detecting meaningful interventional effects.

Adequately performed and high level of evidence studies that report the prevalence of psychological distress for PICS-F are, to our knowledge, relatively scarce. Our study showed that the prevalence of PTSD, anxiety, and depression symptoms in relatives decrease over time, but nonetheless remain high up to 6 months post-ICU discharge. These results are in line with the sparce studies currently available in the field and underline the urge to search for new effective therapies [1–4].

As for potential clinical implications, previous research underscores the importance of clear communication on ICU procedures and relatives are in need of comprehensive information about the ICU treatment. However, such communication is scarcely provided by ICU staff for various reasons and these unmet informational needs may negatively impact mental health [41–49]. Although family satisfaction is often relatively high, a lack of understanding may be present in up to 70% of relatives, and often these relatives find the information provided hard to understand [20, 42, 50]. The current intervention could therefore be a pragmatic solution, enabling relatives of ICU patients to receive accurate and complete information about the ICU environment and treatment using a simple, generalizable intervention without considerably increasing staff workload. Additionally, in the future, when the necessary techniques are deemed ready, the ICU-VR intervention can potentially be supplemented with artificial intelligence (AI), that may enable adaptive interactions to meet specific needs of an individual, which is currently not possible in the ICU-VR intervention [51]. Importantly, relatives appreciated ICU-VR and stated that it helped them understand ICU treatment. The vast majority of our participants indicated that they would recommend ICU-VR to other relatives of ICU patients, suggesting their satisfaction with the intervention.

This study was performed during the beginning of the COVID-19 pandemic, which resulted in some logistical issues, such as hospital visitation regulations. We therefore stratified and randomized participants based on the relatives’ ability to visit the hospital as restricted by COVID-19 regulations. Additionally, a small number (7%) of participants in the intervention group had to receive the first ICU-VR intervention at home, instead of in the hospital.

The strengths of this study are its multicenter design, encompassing both academic and non-academic hospitals, the use of online questionnaires to limit observer bias, the use of validated questionnaires, and the diverse group of included participants, varying in educational levels, gender, relationships to the patient, and types of admission.

Several limitations of this study must be acknowledged. First, at the outset of this study, no prior research was available to define the expected effect estimate, which may have led to our study being underpowered. However, despite the lack of a sample size calculation this is currently one of the studies in its kind that included the most relatives [34, 36]. Second, the inclusion rate was relatively low with 33%. While many potential participants expressed interest in the intervention, they were discouraged by the extensive requirement to complete questionnaires. Nonetheless, we reached our sample size twice a fast than the 30 months required in a recent comparable multicenter intervention study, despite enrolling more than twice the number of participants [34]. Third, the ICU-VR intervention was only available in Dutch language, which may introduce selection bias. This may be particularly relevant considering that non-native speakers are probably in greater need of such an intervention due to their challenges in understanding ICU treatment details due to language barriers and are at risk of receiving less information and emotional support [52]. Fourth, the partly self-composed questionnaires to measure satisfaction and experiences have not been validated, which limits the reliability of these data [31, 32, 53]. Fifth, we did not measure whether the VR intervention resulted in different communication strategies. While VR provides part of the ICU care explanation, potentially reducing the time required for personal contact between staff and relatives, it may lack the human warmth, empathy, and tailored interactions that families often value. Nonetheless, the ratings of the quality of care—encompassing reception, guidance, and information provision—did not differ significantly between groups. Future research should explore whether VR affects the depth and frequency of doctor-relative interactions, as well as the potential impact of these changes on family satisfaction. Sixth, the follow-up rate decreases over time in both groups, leading to a smaller sample size at later time points. This reduction in survey returns may have resulted in a lower power to detect significant effects over time.

## Conclusions

In this multicenter patient-clustered randomized controlled trial, ICU-VR did not improve mental health distress symptoms or HRQoL 6-months after a patients discharge among relatives. The study highlights the significant and persistent psychological impact of ICU admissions on relatives, lasting months post-discharge, and demonstrated that ICU-VR could improve informational satisfaction. Future studies should focus on a multi-faceted approach to improve mental health of relatives of critically ill patients.

## Supplementary Information

Additional file1 (DOCX 1272 KB)

## Abbreviations

ICU: Intensive Care Unit
ICU-VR: Intensive Care Unit-specific Virtual Reality
PICS-F: Post Intensive Care Syndrome Family
HRQoL: Health-related quality of life
VR: Virtual Reality
PTSD: Posttraumatic stress disorder
IES-R: Impact of Event Scale-Revised
HADS: Hospital Anxiety and Depressions Scale
SF-36: Short Form-36
MCS: Mental component score
PCS: Physical component score
SD: Standard deviation
CQI: Consumer Quality Index
RATE-XR: Reporting the early stage clinical evaluation of extended reality-based intervention trials
OR: Odds ratio
AI: Artificial Intelligence
ASD: Acute Stress Disorder

## Declarations

### Availability of data and materials

The data that support the findings of this study are available on request via the corresponding author MvG.
