# Supplementary material for: Effect of an intensive care unit virtual reality intervention on relatives´ mental health distress: a multicenter, randomized controlled trial
Source: Crit Care. 2025 Feb 5;29:62. doi: 10.1186/s13054-025-05281-2 (PMC11796184; doi:10.1186/s13054-025-05281-2)
Supplement: Supplementary file 1 — Additional file1 (DOCX 1272 KB) [file 13054_2025_5281_MOESM1_ESM.docx]

**Supplementary information**

**Title**: **Effect of an Intensive Care Unit Virtual Reality Intervention on relatives´ mental health distress: a multicenter, randomized controlled trial.**

**Journal name**: Critical Care

**Authors**: Denzel L. Q. Drop, MD^1,*^; Johan H. Vlake, BSc^1,2,*;^; Evert-Jan Wils, MD, PhD^2^; Jasper Van Bommel, MD, PhD^1^; Christian Jung, MD, PhD^3,4^; Denise E. Hilling, MD, PhD^5^; O. Joseph Bienvenu, MD, PhD^6^; Tim I.M. Korevaar, MD, PhD^7^; Anna F.C. Schut, MD, PhD^8^; Margo M.C. van Mol, PhD^1^; Diederik Gommers, MD, PhD^1^; Michel E. van Genderen, MD, PhD^11^

*These authors contributed equally to this manuscript.

^1^ Department of Adult Intensive Care, Erasmus MC, University Medical Center Rotterdam, Rotterdam, The Netherlands

^2^ Department of Intensive Care, Franciscus Gasthuis & Vlietland, Rotterdam, The Netherlands

^3^ Medical Faculty and University Hospital of Düsseldorf, Cardiovascular Research Institute Düsseldorf (CARID), Heinrich-Heine University Düsseldorf, 40225 Düsseldorf, Germany

^4^ Department of Cardiology, Pulmonology and Vascular Medicine, Medical Faculty, Heinrich-Heine-University Düsseldorf, Düsseldorf, Germany

^5^ Department of Surgical Oncology and Gastrointestinal Surgery, Erasmus MC Cancer Institute, University Medical Center Rotterdam, Rotterdam, The Netherlands

^6^ Department of Psychiatry and Behavioural Sciences, Johns Hopkins University School of Medicine, Baltimore, Maryland, USA

^7^ Department of Internal Medicine, Division of Vascular Medicine and Pharmacology, Erasmus MC, University Medical Center Rotterdam, Rotterdam, The Netherlands

^8^ Department of Intensive Care, Ikazia Hospital, Rotterdam, The Netherlands

**Corresponding author:**
Michel E. van Genderen
m.vangenderen@erasmusmc.nl

Erasmus MC, University Medical Center Rotterdam
Department of Adult Intensive Care (internal postadress – Room Ne-403)
Doctor Molewaterplein 40, 3015 GD Rotterdam, the Netherlands

| **Table S1 Prevalence of psychological distress in family members – overall cohort and between time points** | | | | |
| --- | --- | --- | --- | --- |
|  | Composite score,  **n (%)** | Anxiety,  **n (%)** | Depression,  **n (%)** | PTSD,  **n (%)** |
| T0 | 69/158 (44) | 65/158 (41) | 48/158 (30) | - |
| T1 | - | - | - | 57/112 (51) |
| T2 | 45/110 (41) | 37/110 (34) | 23/110 (21) | 31/111 (28) |
| T3 | 47/113 (42) | 34/113 (30) | 27/113 (24) | 27/113 (24) |
| T4 | 36/104 (35) | 26/104 (25) | 20/104 (19) | 22/105 (21) |
| OR (95% CI), T0/T1* vs. T2 | 1.20 (0.64 – 2.24) | 0.74 (0.38 – 1.44) | 0.53 (0.24 – 1.18) | 0.09 (0.03 – 0.28) |
| p-value | 0.57 | 0.38 | 0.12 | <0.01 |
| OR (95% CI), T0/T1* vs. T3 | 0.94 (0.51 – 1.74) | 0.50 (0.26 – 0.98) | 0.62 (0.28 – 1.34) | 0.03 (0.01 – 0.11) |
| p-value | 0.84 | <0.05 | 0.22 | <0.01 |
| OR (95% CI), T0/T1* vs. T4 | 0.68 (0.36 – 1.31) | 0.38 (0.19 – 0.77) | 0.40 (0.17 – 0.92) | 0.02 (0.01 – 0.10) |
| p-value | 0.25 | <0.01 | 0.03 | <0.01 |
| OR (95% CI), T2 vs. T3 | 0.73 (0.32 – 1.70) | 0.66 (0.28 – 1.56) | 1.34 (0.32 – 5.56) | 0.17 (0.04 – 0.70) |
| p-value | 0.47 | 0.34 | 0.68 | 0.01 |
| OR (95% CI), T2 vs. T4 | 0.50 (0.20 – 1.21) | 0.47 (0.19 – 1.19) | 0.73 (0.16 – 3.41) | 0.17 (0.04 – 0.76) |
| p-value | 0.12 | 0.11 | 0.69 | 0.02 |
| OR (95% CI), T3 vs. T4 | 0.56 (0.21 – 1.51) | 0.28 (0.04 – 1.87) | 0.57 (0.10 – 3.40) | 0.90 (0.15 – 5.44) |
| p-value | 0.26 | 0.19 | 0.54 | 0.91 |
| T0 (retrospective), T1 (at ICU discharge), T2 (1 month), T3 (3 months), T4 (6 months).  *T0 (retrospective) for the composite score, anxiety and depression, T1 (at ICU discharge) for PTSD. | | | | |

| **Table S2 Symptoms of anxiety, depression, and PTSD in family members – overall cohort and between time points** | | | | | | |
| --- | --- | --- | --- | --- | --- | --- |
|  | Anxiety*,  **median (95% range)** | | Depression**,  **median (95% range)** | | PTSD***,  **median (95% range)** | |
| T0 | 6 (0 – 17) | | 4 (0 – 17) | | - | |
| T1 | - | | - | | 24 (4 – 60) | |
| T2 | 5 (0 – 18) | | 3 (0 – 18) | | 14 (0 – 47) | |
| T3 | 5 (0 – 16) | | 3 (0 – 16) | | 12 (0 – 52) | |
| T4 | 4 (0 – 15) | | 2 (0 – 14) | | 10 (0 – 46) | |
| Difference (95% CI), T0 vs. T2 | -0.14 (-0.91 – 0.63) | | 0.57 (-0.16 – 1.29) | | -7.32 (-9.13 – -5.51) | |
| p-value | 0.72 | | 0.13 | | <0.01 | |
| Difference (95% CI), T0 vs. T3 | -0.91 (-1.67 – -0.14) | | -0.12 (-0.83 – 0.59) | | -9.67 (-11.48 – -7.85) | |
| p-value | 0.02 | | 0.74 | | <0.01 | |
| Difference (95% CI), T0 vs. T4 | -1.46 (-2.25 – -0.67) | | -0.69 (-1.42 – 0.05) | | -10.85 (-12.72 – -8.98) | |
| p-value | <0.01 | | 0.07 | | <0.01 | |
| Difference (95% CI), T2 vs. T3 | -0.71 (-1.26 – -0.16) | | -0.67 (-1.24 – -0.11) | | -2.33 (-3.92 – -0.74) | |
| p-value | 0.01 | | 0.02 | | 0.01 | |
| Difference (95% CI), T2 vs. T4 | -1.13 (-1.69 – -0.57) | | -1.08 (-1.66 – **-**0.49) | | -3.16 (-4.80 – -1.53) | |
| p-value | <0.01 | | <0.01 | | <0.01 | |
| Difference (95% CI), T3 vs. T4 | -0.51 (-1.03 – 0.00) | | -0.45 (-0.97 – 0.08) | | -1.00 (-2.50 – 0.49) | |
| p-value | 0.05 | | 0.10 | | 0.19 | |
| T0 (retrospective), T1 (at ICU discharge), T2 (1 month), T3 (3 months), T4 (6 months).  *Anxiety subscale sum score of the HADS questionnaire, a score ≥8 is considered prevalent anxiety.  **Depression subscale sum score of the HADS questionnaire, a score of ≥ is considered prevalent depression.  ***Sum score of the IES-R questionnaire, a score ≥24 is considered prevalent PTSD. | | | | | | |
| **Table S3 Mental and physical quality of life in family members – overall cohort and between time points** | | | | | |  |
|  | | **Mental QoL**,  median (IQR) | | **Physical QoL**,  median (95% range) | |  |
| T0 | | 49.6 (18.8 – 63.5) | | 55.9 (28.8 – 64.8) | |  |
| T2 | | 44.6 (15.9 – 61.8) | | 55.2 (26.4 – 64.6) | |  |
| T3 | | 46.7 (18.6 – 62.1) | | 55.1 (26.3 – 64.2) | |  |
| T4 | | 51.3 (19.1 – 62.4) | | 55.8 (27.3 – 63.4) | |  |
| Difference (95% CI), T0 vs. T2 | | -6.35 (-8.64 – -4.06) | | -0.20 (-1.47 – 1.07) | |  |
| p-value | | <0.01 | | 0.76 | |  |
| Difference (95% CI), T0 vs. T3 | | -3.97 (-6.23 – -1.71) | | -1.21 (-2.46 – 0.04) | |  |
| p-value | | <0.01 | | 0.06 | |  |
| Difference (95% CI), T0 vs. T4 | | -0.68 (-2.99 – 1.62) | | -0.91 (-2.19 – 0.36) | |  |
| p-value | | 0.56 | | 0.16 | |  |
| Difference (95% CI), T2 vs. T3 | | 2.05 (0.02 – 4.08) | | -0.89 (-2.11 – 0.33) | |  |
| p-value | | 0.05 | | 0.15 | |  |
| Difference (95% CI), T2 vs. T4 | | 5.35 (3.27 – 7.42) | | -0.80 (-2.04 – 0.45) | |  |
| p-value | | <0.01 | | 0.21 | |  |
| Difference (95% CI), T3 vs. T4 | | 3.19 (1.42 – 4.95) | | 0.12 (-0.98 – 1.22) | |  |
| p-value | | <0.01 | | 0.83 | |  |
| T0 (retrospective), T2 (1 month), T3 (3 months), T4 (6 months). | | | | | |  |

| **Table S4a Univariable analysis of demographic and ICU-related risk factors for one or more mental health symptoms in relatives – overall cohort** | | | | | | | | | |
| --- | --- | --- | --- | --- | --- | --- | --- | --- | --- |
|  | | **Mental health symptoms at 1 month** | |  | **Mental health symptoms at 3 months** | |  | **Mental health symptoms at 6 months** | |
|  | | Univariable OR (95% CI) | p-value |  | Univariable OR (95% CI) | p-value |  | Univariable OR (95% CI) | p-value |
| **Family** | |  |  |  |  |  |  |  |  |
| Age | | 0.98 (0.95 – 1.01) | **0.17** |  | 0.98 (0.95 – 1.01) | **0.17** |  | 0.98 (0.95 – 1.01) | **0.13** |
| Gender, Female | | 3.64 (1.51 – 8.78) | **<0.01** |  | 3.82 (1.63 – 8.94) | **<0.01** |  | 3.77 (1.45 – 9.80) | **0.01** |
| Relationship to survivor | |  |  |  |  |  |  |  |  |
|  | Other (ref) |  |  |  |  |  |  |  |  |
|  | Parent | 1.60 (0.48 – 5.34) | 0.45 |  | 2.54 (0.80 – 8.10) | **0.12** |  | 2.68 (0.77 – 9.31) | **0.12** |
|  | Spouse | 2.60 (0.80 – 8.43) | **0.11** |  | 2.50 (0.77 – 8.08) | **0.13** |  | 2.48 (0.69 – 8.86) | **0.16** |
| Educational level^1^ | |  |  |  |  |  |  |  |  |
|  | Lower education (ref) |  |  |  |  |  |  |  |  |
|  | Higher education | 1.24 (0.58 – 2.69) | 0.58 |  | 1.00 (0.47 – 2.12 | 0.99 |  | 0.94 (0.41 – 2.13) | 0.88 |
| Employment status, unemployed | | 0.71 (0.33 – 1.53) | 0.38 |  | 0.91 (0.43 – 1.92) | 0.80 |  | 0.94 (0.42 – 2.13) | 0.88 |
| History of mental illness, no | | 0.36 (0.14 – 0.97) | **0.04** |  | 0.27 (0.10 – 0.71 | **0.01** |  | 0.19 (0.06 – 0.56) | **<0.01** |
| Intervention, control | | 0.99 (0.46 – 2.13) | 0.99 |  | 1.27 (0.60 – 2.70) | 0.53 |  | 0.67 (0.29 – 1.55) | 0.35 |
| **Psychological well-being** | |  |  |  |  |  |  |  |  |
| Anxiety symptoms at ICU admission | | 3.51 (1.50 – 8.22) | **<0.01** |  | 2.85 (1.29 – 6.30) | **0.01** |  | 2.11 (0.91 – 4.89) | **0.08** |
| Depression symptoms at ICU admission | | 3.78 (1.49 - 9.58) | **<0.01** |  | 2.84 (1.20 – 6.72) | **0.02** |  | 2.65 (1.10 – 6.40) | **0.03** |
| Mental HRQoL | | 0.96 (0.92 – 0.99) | **0.02** |  | 0.94 (0.91 – 0.97) | **<0.01** |  | 0.96 (0.93 – 0.99) | **0.02** |
| **ICU patient** | |  |  |  |  |  |  |  |  |
| Age | | 0.99 (0.97 – 1.02) | 0.60 |  | 1.00 (0.97 – 1.03) | 0.99 |  | 1.00 (0.97 – 1.03) | 0.96 |
| Gender, male | | 1.51 (0.69 – 3.34) | 0.30 |  | 2.05 (0.92 – 4.58) | **0.08** |  | 2.37 (0.97 – 5.79) | **0.06** |
| APACHE-IV | | 1.01 (1.00 – 1.02) | **0.20** |  | 1.01 (1.00 – 1.02) | **0.20** |  | 1.01 (0.99 – 1.02) | 0.41 |
| Hospital length of stay | | 1.00 (0.99 – 1.01) | 0.89 |  | 1.00 (0.99 – 1.01) | 0.67 |  | 1.00 (0.98 – 1.01) | 0.43 |
| ICU length of stay | | 1.00 (0.99 – 1.02) | 0.74 |  | 1.00 (0.99 – 1.02) | 0.83 |  | 0.99 (0.97 – 1.02) | 0.49 |
| Mechanical ventilation | | 1.44 (0.41 – 5.10) | 0.57 |  | 0.84 (0.24 – 2.93) | 0.79 |  | 0.77 (0.20 – 2.94) | 0.71 |
| COVID-19 diagnosis, no | | 1.91 (0.75 – 4.85) | **0.17** |  | 0.68 (0.30 – 1.57) | 0.37 |  | 0.70 (0.30 – 1.63) | 0.41 |
| ICU mortality | | 1.48 (0.28 – 7.67) | 0.64 |  | 1.85 (0.47 – 7.28) | 0.38 |  | 2.03 (0.55 – 7.55) | 0.29 |
| ^1^ Higher vocational education and academic education were classified as higher education compared to lower education levels. | | | | | | | | | |

| **Table S4b Multivariable analysis of demographic and ICU-related risk factors for one or more mental health symptoms in relatvies – overall cohort** | | | | | | | | | |
| --- | --- | --- | --- | --- | --- | --- | --- | --- | --- |
|  | | **Mental health symptoms at 1 month** | |  | **Mental health symptoms at 3 months** | |  | **Mental health symptoms at 6 months** | |
|  | | Multivariable OR (95% CI) | p-value |  | Multivariable OR (95% CI) | p-value |  | Multivariable OR (95% CI) | p-value |
| **Family** | |  |  |  |  |  |  |  |  |
| Age | | 0.97 (0.93 – 1.02) | 0.21 |  | 2.00 (0.96 – 1.05) | 0.87 |  | 0.98 (0.94 – 1.02) | 0.32 |
| Gender, Female | | 3.41 (1.22 – 9.54) | **0.02** |  | 2.04 (0.74 – 5.60) | 0.17 |  | 2.03 (0.68 – 6.09) | 0.21 |
| Relationship to survivor | |  |  |  |  |  |  |  |  |
|  | Other (ref) |  |  |  |  |  |  |  |  |
|  | Parent | 1.37 (0.29 – 6.50) | 0.69 |  | 3.26 (0.65 – 16.37) | 0.15 |  | 1.59 (0.35 – 7.21) | 0.55 |
|  | Spouse | 2.07 (0.45 – 9.58) | 0.35 |  | 2.90 (0.68 – 12.41) | 0.15 |  | 2.18 (0.52 – 9.10) | 0.29 |
| History of mental illness, no | | 0.63 (0.19 – 2.12) | 0.46 |  | 0.41 (0.13 – 1.27) | 0.12 |  | 0.23 (0.07 – 0.81) | **0.02** |
| **Psychological well-being** | |  |  |  |  |  |  |  |  |
| Anxiety or depression symptoms at ICU admission | | 4.99 (1.57 – 15.83) | **<0.01** |  | 2.23 (0.76 – 6.58) | 0.15 |  | 1.28 (0.39 – 4.19) | 0.69 |
| Mental HRQoL | | 1.01 (0.96 – 1.06) | 0.83 |  | 0.97 (0.91 – 1.00) | 0.06 |  | 0.98 (0.93 – 1.02) | 0.31 |
| **ICU patient** | |  |  |  |  |  |  |  |  |
| Gender, male | | - | - |  | 1.78 (0.66 – 4.78) | 0.25 |  | 2.47 (0.87 – 7.06) | 0.09 |
| APACHE-IV | | 1.01 (0.99 – 1.02) | 0.34 |  | 1.01 (0.99 – 1.02) | 0.48 |  | - | - |
| COVID-19 | | 2.03 (0.67 – 6.17) | 0.21 |  | - | - |  | - | - |
| ^1^ Higher vocational education and academic education were classified as higher education compared to lower education levels. | | | | | | | | | |

| 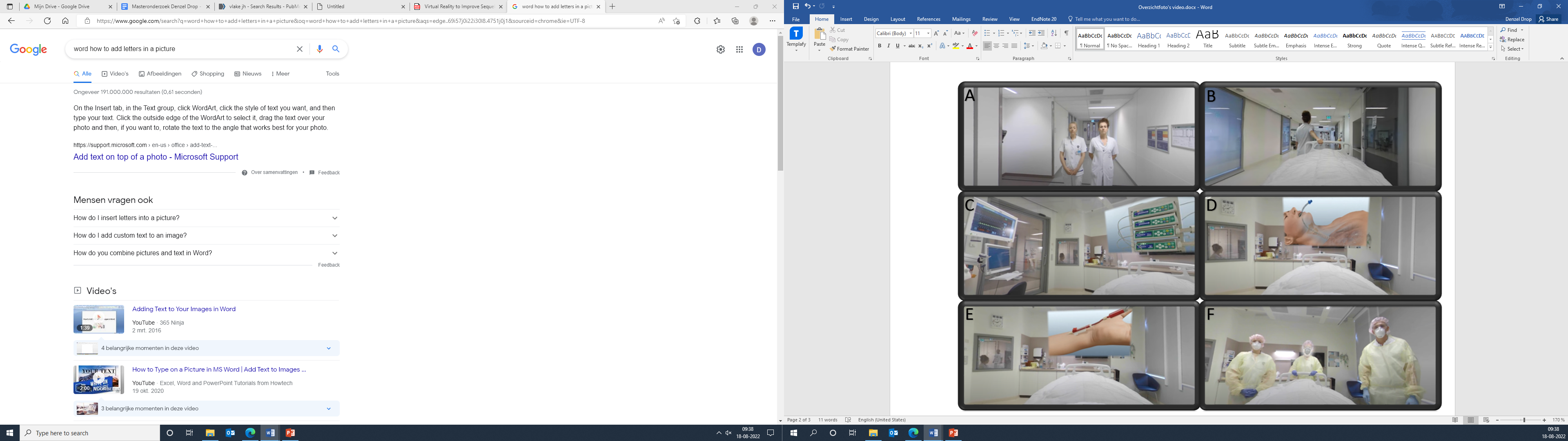 |
| --- |
| **Suppl Fig 1** The Intensive Care Unit-specific Virtual Reality (ICU-VR) intervention  **A-F** Images of the ICU-VR video with (**A**) an introduction by an intensivist and ICU nurse to welcome the relative to the ICU-VR environment, (**B**) explaining daily movements at an ICU and the route to the ICU room, (**C**) explanation of monitors and noises in an ICU room, (**D**) information regarding airway and breathing management such as intubation and tracheal tube suction, (**E**) information and necessity of central/peripheral lines, (**F**) information and necessity of the treatment team, ICU workflow and personal protection equipment. |
